# Transplantation of a Testicle from the Groin to the Scrotum

**Published:** 1880-12

**Authors:** 


### TRANSPLANTATION OF A TESTICLE FROM THE GROIN TO THE SCROTUM.

Wood, of London, reports the following case: The patient was aged thirteen. A tumor, which disappeared when lying down, was noticed in the right groin, when he was quite young. For this he had worn a truss all his life. This prevented the descent of the tumor until ten days before he was seen by Mr. Wood, when it slipped past the instrument and could not be returned. This was followed in four days by pain, rapid increase in size, sickness and constipation. When examined by Mr. Wood, there was found at the right external ring, a solid, irreducible, excessively painful tumor, that gave no impulse on coughing. The right testicle was absent from the scrotum. Diagnosis: Inflamed, undescending testicle. An ice-bag was applied, and in a week the tumor had been reduced to its normal size. It could not, however, be returned to the abdominal cavity. Mr. Wood now exposed the testicle by an incision over the abdominal ring, and found it smaller than its fellow. The cavity of the *tunica vaginalis* could not be found, and appeared to be obliterated. The testicle was attached to the pillars of the ring by very firm adhesions, which were with difficulty broken down. By freeing the cord for about an inch and a half, he was able to bring the testicle down about an inch, though he found the cord considerably shortened. He then everted the scrotum, stitched the testicle to the everted part with catgut and sewed up the opening, putting in a drainage tube, with a pad above the testicle, all being done under antiseptic precautions. The patient did well, the testicle remaining outside of the ring, and the temperature never going above 99°. A water-pad truss was worn to keep the gland away from the ring, when the patient was discharged.—*Lancet.*

